# Effects of Anti-Cytokine Antibodies on Gut Barrier Function

**DOI:** 10.1155/2019/7028253

**Published:** 2019-11-12

**Authors:** Fang Liu, Seul A. Lee, Stephen M. Riordan, Li Zhang, Lixin Zhu

**Affiliations:** ^1^Department of General Surgery and Central Lab, The First Affiliated Hospital of Anhui Medical University, Hefei 230022, China; ^2^School of Biotechnology and Biomolecular Sciences, University of New South Wales, Sydney, NSW 2052, Australia; ^3^Gastrointestinal and Liver Unit, Prince of Wales Hospital, University of New South Wales, Sydney, NSW, Australia

## Abstract

Anti-cytokine antibodies are used in treating chronic inflammatory diseases and autoimmune diseases such as inflammatory bowel disease and rheumatic diseases. Patients with these diseases often have a compromised gut barrier function, suggesting that anti-cytokine antibodies may contribute to the re-establishment of gut barrier integrity, in addition to their immunomodulatory effects. This paper reviews the effects of anti-cytokine antibodies on gut barrier function and their mechanisms.

## 1. Introduction

Cytokines are a large group of proteins produced by various immune and non-immune cells, which are key signalling molecules regulating the immunity. Increased levels of proinflammatory cytokines are hallmarks of chronic inflammatory diseases and autoimmune diseases. Enhanced expressions of proinflammatory cytokines in mucosal tissues such as interleukin- (IL-) 1*β*, IL-6, IL-8, tumor necrosis factor- (TNF-) *α*, and interferon- (IFN-) *γ* are found in patients with inflammatory bowel disease (IBD). IBD is a chronic inflammatory condition caused by inflammatory cytokines or bacterial species directly damaging the intestinal epithelial barrier [1–3]. Similarly, in patients with psoriasis, elevated levels of proinflammatory cytokines such as IL-1*β*, IL-2, IL-6, IL-8, TNF-*α*, and IFN-*γ* have been detected in psoriatic lesional skin as compared to non-lesional and healthy skin and serum levels of the subset of these cytokines are correlated with disease severity [4, 5]. In patients with rheumatoid arthritis, numerous cytokines such as TNF-*α*, IL-1*β*, IL-6, IL-8, and IFN-*γ* are produced and are functionally active in synovial tissues, leading to cytokine-driven joint inflammation and damage [6]. Ankylosing spondylitis is a chronic inflammatory condition of the spine, and serum levels of various cytokines have been found at increased levels in patients [7]. Due to the importance of cytokines in the pathogenesis of these diseases, anti-cytokine antibodies have been used as treatment strategies to restore the immune homeostasis.

Increased intestinal permeability, also referred to as leaky gut, is often seen in chronic inflammatory conditions mentioned above. Previous studies have shown that some cytokines are able to directly damage the gut barrier function. Given this, it is possible that anti-cytokine antibodies may contribute to their clinical beneficial effects by modulating the gut barrier function in addition to regulating the immune responses. Here, we review the effects of clinically used anti-cytokine antibodies on gut barrier function and their associated mechanisms.

## 2. Clinically Used Anti-Cytokine Antibodies

Anti-cytokine antibodies neutralise cytokine activities by blocking the interaction between cytokines and their receptors. Anti-cytokine antibodies currently approved by the Food and Drug Administration for clinical uses are listed in Table 1. Anti-TNF-*α* and anti-p40 subunit antibodies are used for the treatment of IBD. Currently, four anti-TNF-*α* antibodies are available including infliximab, adalimumab, certolizumab, and golimumab. Infliximab and adalimumab are able to neutralise soluble, transmembrane, and receptor-bound TNF-*α*, while certolizumab and golimumab are able to neutralise soluble and transmembrane forms of TNF-*α* [8–10]. The use of anti-TNF-*α* antibodies to treat IBD has been implicated for more than two decades, and anti-TNF-*α* antibodies were useful in patients who are steroid unresponsive [11, 12]. Ustekinumab, a human monoclonal antibody that targets the shared p40 subunit of IL-12 and IL-23, has also been approved for treating CD.

A number of cytokine neutralising antibodies have been approved in treating psoriasis, a chronic inflammatory skin disease. The candidate targets for such therapeutic antibodies include TNF-*α* (infliximab, adalimumab, and certolizumab), IL-12 (ustekinumab), IL-23 (ustekinumab, guselkumab, and tildrakizumab), and IL-17 (ixekizumab, secukinumab, and brodalumab). Secukinumab which targets IL-17A/F is also used to treat ankylosing spondylitis and psoriatic arthritis.

Rheumatoid arthritis, a chronic inflammatory disease affecting the joints, is another disease being treated with anti-cytokine therapies [13]. Therapeutic targets, TNF-*α* and IL-6, are the two key cytokines involved in the pathogenesis of the disease. Tocilizumab and sarilumab, both targeting IL-6 receptors, have been approved for treating rheumatoid arthritis.

## 3. Increased Intestinal Permeability in Diseases Treated with Anti-Cytokine Antibodies

Previous studies found that patients with chronic inflammatory and autoimmune diseases mentioned above usually have a compromised gut barrier function, indicated by abnormal permeation of non-metabolised sugar molecules, radioisotopes, and polyethylene glycols (Table 2). The leaky gut allows enhanced passages of luminal bacterial species and bacterial products such as toxins and antigens and is believed to contribute to the pathogenesis of chronic inflammatory and autoimmune diseases [14–16].

Patients with IBD, including both Crohn's disease (CD) and ulcerative colitis (UC) which are the two major clinical forms of the disease, are known to have increased intestinal permeability, and increased intestinal permeability is frequently observed even in patients under remission. Some studies have reported abnormally enhanced intestinal permeability in relatives and spouse of patients with IBD, suggesting that genetic and environmental factors also play a major role in shaping the gut barrier integrity (Table 2). Additionally, elevated intestinal permeability has also been reported in patients with psoriasis, rheumatoid arthritis, and ankylosing spondylitis (Table 2).

## 4. Effects of Clinically Used Anti-Cytokine Antibodies on Gut Barrier Function

### 4.1. Tumor Necrosis Factor-*α*

TNF-*α* exerts damaging effects on the intestinal epithelial barrier by affecting the tight junction functions [17]. Schmitz et al. showed that the transepithelial electrical resistance was significantly reduced in intestinal epithelial HT-29/B6 cells following TNF-*α* incubation, indicating an increased intestinal permeability. Additionally, the tight junction complexity was decreased, indicated by a reduction in the number of strands from 4.7 to 3.4 and this effect was alleviated by the presence of tyrosine kinase inhibitor genistein and protein kinase A inhibitor H-8 [18]. Inhibition of TNF-*α*-induced apoptosis did not improve the permeability defects, further confirming its primary influence on the junctional barrier. The structural and functional changes in the tight junctions were found to be potentially caused by an increased expression of myosin light chain kinase (MLCK) protein, as the inhibition of MLCK expression was able to prevent the intestinal permeability defects induced by TNF-*α* [19].

In addition to the intestinal pathology caused by the epithelial responses towards TNF-*α*, the contribution of intestinal epithelial cell-derived TNF in disease pathogenesis has also been studied. Chronic epithelial production of TNF may initiate CD-like pathology, and this has been demonstrated by Roulis et al. Selective chronic overexpression of TNF by intestinal epithelial cells led to early activation of underlying mesenchymal cells and could successfully cause full development of Crohn-like pathology in mice [20].

#### 4.1.1. Anti-TNF-*α* Antibodies

The principal mechanism of action of infliximab is to neutralise TNF-*α* activity (Table 3). Infliximab was also found to downregulate IFN-*γ* production in colonic T cell cultures isolated from patients with CD [21]. Additionally, a number of studies have demonstrated that infliximab induced apoptosis in a subpopulation of inflammatory cells that are critical for the perpetuation of inflammation, such as monocytes and lamina propria T lymphocytes [22, 23]. Mucosal T lymphocytes in CD display defective apoptosis, and infliximab may reverse the apoptotic resistance in lamina propria T lymphocytes by inducing cell death, hence restoring a balanced immune response [24].

*(1) Anti-TNF-α Antibodies and the Gut Barrier*. Increased intestinal permeability has been considered as a pathogenic event in CD. Patients with CD usually have increased intestinal permeability which leads to enhanced translocation of gut commensal microbes and their products, ultimately resulting in chronic inflammation. Suenaert et al. studied the intestinal permeability in active CD patients and healthy individuals by measuring permeation of ^51^Cr-EDTA through small intestine prior and post infliximab application. The study found that the increased intestinal permeability observed in patients before treatment was significantly reduced to levels within the range of those found in healthy individuals [25]. A similar finding was reported by Noth et al. They measured differential intestinal uptake of lactulose and mannitol and found that infliximab was able to improve impaired intestinal barrier function in CD patients [26]. These studies indicate that infliximab was able to restore gut barrier function by improving intestinal permeability. However, the restored barrier function might due to the healing of the injured mucosa; hence, whether infliximab has a direct protective effect on the intestinal epithelium requires further research.

The direct effects of infliximab on epithelial apoptosis have been studied using human intestinal biopsies and animal models (Table 3). Zeissig et al. found that the level of epithelial apoptosis was reduced in biopsies obtained from patients with chronic active CD following infliximab treatment; however, expression of tight junction proteins including occludin, and claudin-1/4 was not affected by anti-TNF-*α* antibody therapy [27]. Another research group also reported that the anti-TNF-*α* antibody caused significant suppression of intestinal inflammation and epithelial cell apoptosis in a murine model with Crohn's-like ileitis [28]. The membrane-bound expression of the death receptor Fas/CD95 in intestinal epithelial cells was reduced following TNF-*α* neutralisation, paralleled with an increase in lamina propria mononuclear cell apoptosis. Furthermore, by using a rat model with indomethacin-induced enterocolitis, Cury et al. showed that prelesion administration of infliximab markedly reduced the intestinal permeability without causing microscopic or macroscopic alterations [29]. By using the same model, Colpaert et al. showed that alleviation of small bowel inflammation following TNF neutralisation was partially due to the improvement of the increased intestinal permeability [30]. These studies collectively suggest a beneficial role of anti-TNF-*α* therapy in protecting intestinal epithelial cells from apoptosis and improving intestinal epithelial permeability.

*(2) Anti-TNF-α Antibodies and Microbiota*. In comparison to thiopurine monotherapy, anti-TNF monotherapy as a treatment for IBD was associated with increased risks of mycobacterial and bacterial infection, however with a reduced risk of opportunistic viral infection [31]. Cytokines secreted by the host could be employed by some bacteria as growth factors. An *in vitro* study showed that the growth of three common nosocomial pathogens *Staphylococcus aureus*, *Acinetobacter* spp., and *Pseudomonas aeruginosa* was favoured by TNF-*α*, IL-1*β*, and IL-6 in a concentration-dependent manner, and these effects were attenuated by cytokine-specific antibodies [32]. The same group also reported that the intracellular growth of the tested bacterial species increased significantly when monocytes were primed with high concentrations of TNF-*α*, IL-1*β*, and IL-6 [33]. Another study by Lee et al. found that although TNF-*α* was able to increase the *in vitro* growth of *Escherichia coli*, *in vivo*, this effect was only apparent in neutropenic mice due to the effect being compensated by the recruited neutrophils [34]. These studies indicate that, under some circumstances, cytokines could serve as growth factors and promote the growth of certain microbes; nevertheless, the exact mechanisms on how these cytokines or cytokine inhibitors act on microbial growth have not been studied in detail.

Retrospective studies examining the gut microbiota composition before and during infliximab treatment have provided predictive biomarkers for therapeutic response. Rajca et al. found that low abundance of *Firmicutes*, particularly *Clostridium coccoides*, *C*. *leptum*, and *Faecalibacterium prausnitzii*, was associated with patients with CD as compared with healthy individuals [35]. More importantly, the lower rate of *Firmicutes* such as *F*. *prausnitzii* and *Bacteroides* was predictive of CD relapse after infliximab withdrawal. Another study examining faecal microbiota from patients with CD and UC also found that a reduced abundance of *Firmicutes*, particularly *Clostridiales*, was strongly correlated with IBD severity, and this decreased level of *Clostridiales* was restored to the level of healthy individuals in patients who responded to infliximab therapy [36]. Similarly, Wang et al. reported that infliximab was able to shift CD-associated microbiota dysbiosis towards a healthy status in paediatric CD patients [37].

*F. prausnitzii* has been considered as an anti-inflammatory commensal bacterium due to its ability in inhibiting the production of proinflammatory cytokines and promoting the productions of anti-inflammatory cytokines [38]. Sokol et al. used an animal model of 2,4,6-trinitrobenzenesulfonic acid- (TNBS-) induced colitis to show that oral administration of either viable *F*. *prausnitzii* bacterial cells or their supernatant was able to restore the microbiota dysbiosis [38]. Reduction in the abundance of *F*. *prausnitzii* correlated with a higher rate of IBD relapse; therefore, *F*. *prausnitzii* has the potential to be used as a probiotic in IBD management [38, 39]. The above microbiome analyses of faecal specimens from IBD patients undergoing infliximab treatment suggest a beneficial role of infliximab in restoring gut microbial dysbiosis.

### 4.2. Interleukin-6

IL-6 is a pleiotropic cytokine with both proinflammatory and anti-inflammatory properties [40]. The signalling pathway of IL-6 could be divided into classic signalling and trans-signalling. The classic signalling pathway is activated by interaction between IL-6 and its receptor expressed on the surface of the target cells, followed by coupling of the signalling receptor gp130, whereas the trans-signalling pathway is activated by a complex of IL-6 and its soluble IL-6 receptor. The anti-inflammatory and regenerative activity of IL-6 is mediated by classic signalling, while the proinflammatory activity is mediated by trans-signalling [40, 41]. Intestinal epithelial cells release IL-6 in response to cytokine and toll-like receptor 4 activation induced by infection, disease, or injury [42].

IL-6 exerts both damaging and protective effects on the intestinal epithelium. IL-6 receptor is found to be expressed on both the apical and basolateral compartments of the intestinal epithelial cells [43, 44]. By using Caco-2 cells as an intestinal barrier model, Suzuki et al. showed that IL-6 was able to enhance the expression of tight junction protein claudin-2, resulting in an increase in cation-selective tight junction permeability without affecting the cell viability [44]. Wang et al. had shown that nuclear factor kappa B (NF-*κ*B) transcription factor, one of the central mediators of intestinal inflammation, could be activated through the stimulation of both basolateral and, to a lesser extent, apical IL-6 [45]. Furthermore, IL-6 also induced polarised expression of intercellular adhesion molecule- (ICAM-) 1, an adhesion molecule that is involved in neutrophil transmigration across the mucosal epithelium during inflammatory responses [46, 47].

IL-6 is known to have protective effects on intestinal epithelial apoptosis [48–50]. Grivennikov et al. demonstrated that IL-6 accelerated the proliferation of tumor-initiating cells and protected premalignant intestinal cells from apoptosis. Furthermore, these effects were mainly driven by the signal transducer and activator of transcription- (STAT-) 3, the pathway that is known to contribute to the pathogenesis of IBD and tumorigenesis of different types of cancers [48]. Although IL-6 is involved in the promotion of tumorigenesis, its protective effects are suggested to be necessary for the maintenance of the intestinal epithelial integrity during tissue injury [51, 52].

#### 4.2.1. Anti-IL-6 Receptor Antibody and Anti-IL-6 Antibody

IL-6 has been used as a therapeutic target, and accumulated evidence has shown that neutralisation of IL-6 activity by the anti-IL-6 antibody and the anti-IL-6 receptor antibody exerts protective effects on the intestinal barrier function (Table 3). The anti-IL-6 receptor monoclonal antibody tocilizumab has been developed to block the trans-signalling of IL-6 activity, and it has been used as an immunosuppressive agent in treating chronic inflammatory diseases such as rheumatoid arthritis, juvenile idiopathic arthritis, and Castleman disease [53]. It has also been used in a pilot clinical trial to treat patients with CD and was found to be beneficial in treating active CD [54].

*(1) Anti-IL-6 Receptor Antibody and Gut Barrier*. Neutralisation of IL-6 activity has several beneficial effects on the gut barrier such as reducing the levels of IL-6 and other proinflammatory cytokines, improving colonic permeability, modulating expressions of tight junction proteins and adhesion molecules, and ameliorating mucosal inflammation (Table 3). These effects have been studied using intestinal epithelial cell lines and different murine models. By using colon carcinoma cell line SW480, Hsu et al. showed that IL-6 was able to promote the clonogenic growth of SW480 cells, indicated by a significant increase of colonies on soft agar as compared with untreated cells. Furthermore, IL-6 also enhanced the invasiveness of SW480 cells, indicated by an increased number of cells invading the Matrigel membrane within the invasion chamber and these effects were blocked when cells were co-incubated with the anti-IL-6 receptor antibody. Additionally, the anti-IL-6 receptor antibody also inhibited the release of matrix metalloproteinase- (MMP-) 9 and MMP-2 by SW480 cells, which are proteases associated with tumor invasiveness in colorectal cancer [55].

The role of the anti-IL-6 receptor antibody has been studied in a T cell transfer murine model of colitis that resembles human CD [56]. Yamamoto et al. showed that mice treated with the anti-IL-6 receptor antibody displayed a lower colitis score as compared with the control animals. Colonic mRNA levels of IFN-*γ*, TNF-*α*, and IL-1*β* were reduced following treatment. Furthermore, colonic expressions of ICAM 1 and vascular cell adhesion molecule (VCAM) 1 were markedly suppressed by the treatment, resulting in diminished leukocyte recruitment and increased T cell apoptosis [57].

A pilot clinical trial examining the efficacy of anti-IL-6 receptor antibody MRA showed a clinical response of 80% in patients with CD as compared to 30% in the placebo group. However, the treatment showed no effect on mucosal healing [54].

*(2) Anti-IL-6 Antibody and Gut Barrier*. The beneficial impact of the anti-IL-6 antibody on the gut barrier has been demonstrated by studies using different animal models. Zahs et al. have used a murine model to study the role of IL-6 in intestinal barrier function during ethanol and burn injury [58]. They found that following damages induced by ethanol and burn injury, anti-IL-6 antibody treatment reduced the morphological changes in the ileum, bacterial translocation to the mesenteric lymph node, and phosphorylation of the myosin light chain in the intestinal epithelial cell as compared with injury alone. Ethanol and burn injury induced a dramatic decrease in the expressions of tight junction protein zonula occluden- (ZO-) 1 and occludin, while mice treated with the anti-IL-6 antibody maintained ZO-1 and occludin localisation with actin. By using a murine model of sepsis with caecal ligation and puncture inducing colonic barrier dysfunction, Nullens et al. showed that the damaging effects on the barrier function were counteracted when the animal was preventively treated with the anti-IL-6 antibody, coinciding with reduced serum levels of IL-6 and IL-10 and colonic level of TNF-*α* [59]. They also found that the mRNA levels of cell adhesion molecules E-cadherin and desmoglein-2 were significantly increased in septic animals and the administration of anti-IL-6 antibody inhibited the upregulated expressions of these proteins, which explains the improved colonic permeability following anti-IL-6 antibody treatment. However, they did not observe a decrease in bacterial translocation towards the bloodstream and mesenteric lymph node, possibly due to the difference of the animal model being used.

Dextran sulfate sodium- (DSS-) induced colitis is widely employed as a murine model of IBD. The cytokine expression profile and histological changes found in acute DSS-induced colitis resemble those observed in human IBD, especially UC [60–62]. DSS induces direct damages on the colonic epithelial cells, leading to increased intestinal permeability and allowing translocation of luminal bacterial species and their products to the submucosal tissue [63–69]. A characteristic of DSS-induced colitis is the momentous infiltrates in the inflammatory lesions, composing mainly of T and B lymphocytes, macrophages, and neutrophils that produce a variety of proinflammatory cytokines such as TNF-*α*, IL-6, IL-8, IL-12, IL-17, and IFN-*γ* [62, 70]. By using a mouse model, Xiao et al. showed that the anti-IL-6 antibody was able to neutralise the increased serum level of IL-6 induced by DSS [71]. Mice treated with the anti-IL-6 antibody also displayed reduced mucosal damages and inflammatory infiltrate. Furthermore, the IL-6 antibody significantly improved the deteriorated intestinal permeability induced by DSS, paralleled with the downregulation of claudin-2 expression. Collectively, these different *in vivo* models have consistently demonstrated the protective effects of the anti-IL-6 antibody on gut barrier function.

### 4.3. Interleukin-12 and Interleukin-23

IL-12 and IL-23 are heterodimers consisting of the p35 and p19 subunit, respectively, and both share the same p40 subunit [72]. IL-12 induces naïve CD4+ T cell differentiation into Th1 cells, which leads to the production of IFN-*γ*, whereas IL-23 induces CD4+ T cell differentiation into Th17 cells leading to the production of IL-17, IL-17F, IL-6, and TNF-*α* [72–75]. Patients with CD manifested increased secretion of both IL-12 and IL-23 as compared with control individuals [76].

#### 4.3.1. Anti-IL-12 Antibody

The beneficial effects of the anti-IL-12 antibody in animal models of enterocolitis have been demonstrated by a number of *in vivo* studies. Davidson et al. showed that the administration of the anti-IL-12 monoclonal antibody successfully inhibited the established colitis in IL-10-deficient mice by blocking IL-12-mediated Th1 development and IFN-*γ* production [77]. In a murine model of TNBS-induced chronic colitis, the administration of the anti-IL-12 antibody exerted improvement of clinical and histological features which led to the abrogation of the established colitis, due to enhanced apoptosis of Th1 cells in the lamina propria and spleen [78, 79]. Furthermore, the stimulation of lamina propria CD4+ T cells isolated from mice treated with the anti-IL-12 antibody failed to secrete IFN-*γ*. These promising results led to the hope of using the anti-IL-12 antibody as a therapeutic agent in treating patients with active CD. A later clinical trial study showed that in patients with improved clinical conditions following anti-IL-12 antibody treatment, reduction in IL-12, IFN-*γ*, and TNF-*α* production by mononuclear cells from the colonic lamina propria was observed [80] (Table 3). However, no significant difference in remission rates has been observed between the treatment group and the placebo group.

#### 4.3.2. Anti-IL-23 Antibody

IL-23 is involved in the initiation and perpetuation of both innate and T cell-mediated intestinal inflammation as shown by studies employing different animal models of colitis [81]. Wang et al. found that the colonic mucosal expression of claudin-8 is significantly diminished in mice with TNBS induced colitis, and addition of anti-IL-23 antibody was able to restore claudin-8 expression which led to the recovery of colitis [82]. Furthermore, Yen et al. showed that IL-23 was responsible for T cell activation and production of proinflammatory mediators IL-17 and IL-6 in IL-10-deficient animal model that spontaneously develops enterocolitis. In addition, they also showed that recombinant IL-23, rather than IL-12, induced exacerbation of colitis [83]. This finding is further supported in a study by Kullberg et al., where the CD4+ T cell adoptive transfer model of colitis showed that IL-23-deficient recipient mice displayed attenuation of colitis [84].

Blockade or genetic ablation of IL-23 was found to attenuate intestinal inflammation and reduce the production of proinflammatory cytokines including TNF-*α*, IFN-*γ*, monocyte chemoattractant protein- (MCP-) 1, IL-6, IL-1*β*, and keratinocyte chemoattractant (KC) [81]. Therapeutic antibody risankizumab that selectively block IL-23 activity was developed and has been shown to be successful in clinical improvement of patients with moderate to severe plaque psoriasis [85–87]. In a more recent study, risankizumab has been investigated to treat patients with active CD and it was found to be more effective than placebo for inducing clinical remission, accompanied with reductions of biomarkers such as serum C-reactive protein and faecal calprotectin [88]. The endoscopic remission induced by risankizumab was associated with decreased colonic expression of genes associated with the IL-23/IL-17 axis such as IL-23, IL-26, and IL-17A [89].

#### 4.3.3. Anti-p40 Antibody

Due to the presence of the common p40 subunit in both IL-12 and IL-23, an antibody against p40 has been developed to target both of the cytokines simultaneously and block the IL-12/23 pathway. Ustekinumab, a human monoclonal antibody targeting the shared p40 subunit of IL-12 and IL-23, has been used to induce and maintain remission in patients with CD. A clinical study conducted by Sandborn et al. showed that ustekinumab induced a clinical response in patients with moderate to severe CD, particularly in those who have previously received infliximab [90]. A later study by the same group reported that patients with moderate to severe CD who were resistant to anti-TNF therapy had increased response rate to induction with ustekinumab as compared with the placebo group [91]. Furthermore, patients with an initial response also had an increased rate of clinical remission.

#### 4.3.4. Anti-p40 Antibody and Gut Microbiota

Microbiome study showed that higher baseline abundance of bacterial species including *Faecalibacterium*, *Bacteroides*, and *Ruminococcus* and lower abundance of *Escherichia* and *Shigella* were positively associated with the therapeutic response of ustekinumab in patients with CD [92]. Moreover, ustekinumab treatment was found to cause alteration of the microbiome community, and changes in microbiota after ustekinumab treatment were able to distinguish subjects in remission from those with active CD. Bacterial species belonged to *Faecalibacterium*, *Blautia*, *Clostridium*, *Ruminococcus*, and *Roseburia* were present at higher abundance in patients in remission than those with active disease.

### 4.4. Interleukin-17

In addition to Th1 and Th2 immune responses, the other sub-lineage of T cell Th17 activated and maintained by IL-23 has been considered to contribute to the perpetuation of IBD, particularly CD. IL-17 is the major cytokine produced by Th17 cells. The Th17 family comprises IL-17A, IL-17B, IL-17C, IL-17D, IL-17E, and IL-17F, of which IL-17A and IL-17F share the highest sequence homology and also many effector functions [16, 93, 94]. Th17 cells are found to play a crucial role in mediating mucosal immune responses against extracellular pathogens [95, 96].

Examination of the chemokine expression profile in HT-29 cells showed that upon IL-17 stimulation, the expression of Th1 recruiting chemokines decreased, and simultaneously, the levels of chemokines specific for Th17 and other immune cell types were increased, suggesting a positive feedback of Th17 recruitment at the site of inflammation [97]. In addition to its proinflammatory role, IL-17 also has a protective role in maintaining intestinal epithelial integrity. Lee et al. showed that IL-17A regulated the localisation of occludin within the crypts of colonic epithelium during DSS-induced injury. It was also found that IL-17R-dependent activation of adaptor protein Act-1 in epithelial cells prevented excess inflammation [98]. Furthermore, IL-17 was shown to promote angiogenesis and colorectal tumorigenesis in human colorectal cancer cells and ablation of IL-17A significantly reduced tumor development in mice [99–101].

#### 4.4.1. IL-17 Blockade

Although believed to contribute to the etiopathology of CD, *in vivo* studies using animal models of colitis have elaborated controversial results regarding the consequences of IL-17 inhibition (Table 3). Ito et al. showed that in the DSS model, only faint manifestations of colitis could be observed in IL-17A knockout mice without gross disruptions of the intestinal epithelium and with only marginal changes of inflammatory infiltrates [102]. However, Yang et al. revealed contrasting results in which they showed that IL-17F-deficient animal represented reduced colitis induced by DSS, whereas IL-17 knockout mice developed more severe colitis [16]. By using the same DDS-induced colitis model, Ogawa et al. showed that treatment with the anti-IL-17 monoclonal antibody enhanced the colitis severity, with elevated mucosal infiltrates and increased mucosal mRNA expressions of TNF-*α*, IFN-*γ*, IL-6, RANTES (CCL5), and IFN-*γ*-induced protein-10 [103]. Similarly, Maxwell et al. showed that in a multidrug resistance mouse model of colitis, exacerbation was associated with IL-17A or IL-17RA inhibition, accompanied with a severe weakening of the intestinal epithelial barrier and augmented colonic inflammation [104]. These studies suggest that IL-17 may rather have a protective role in colitis development.

In contrast to IL-17A or IL-17F inhibition, concurrent blockade of both IL-17A and IL-17F appeared to have a more consistent consequence. In a T cell transfer-induced colitis model, Th17 cell development was correlated with colitis progression and simultaneous neutralisation of both IL-17A and IL-17F was found to be efficient in reducing intestinal inflammation; on the other hand, neutralisation of individual IL-17A or IL-17F was insufficient [105]. These results are supported by another study showing that Th17 cells induced intestinal inflammation via redundant effects of IL-17A and IL-17F [106]. These studies therefore suggest the therapeutic potential of combined IL-17A and IL-17F blockade in treating IBD.

A number of clinical studies have shown that antibodies targeting IL-17 or IL-17 receptors are not suitable in treating CD. Hueber et al. showed that secukinumab, an anti-IL-17A monoclonal antibody, induced serious adverse events when used in patients with active CD and the unfavourable responses were driven in patients with increased inflammatory markers such as C-reactive protein and faecal calprotectin [107]. Furthermore, brodalumab, an anti-IL-17 receptor antibody, was also shown to worsen CD symptoms in patients with active CD [108]. Anti-IL-17 therapy has also been used in treating psoriasis. The exacerbations of IBD in clinical trials of using anti-IL-17 and anti-IL-17R antibodies to treat psoriasis have been recently reviewed [109].

## 5. Conclusion

**Figure 1:**
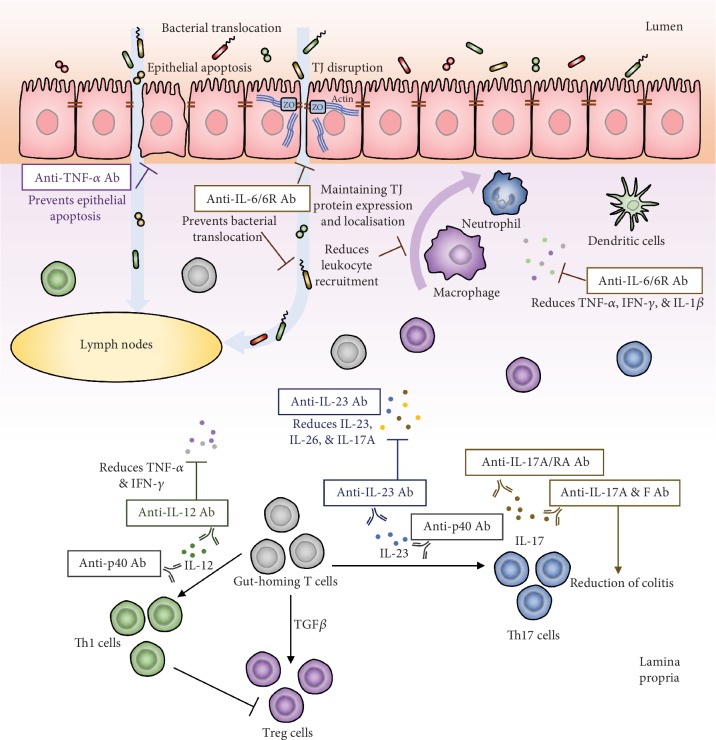
Summary of mechanisms of anti-cytokine antibodies on restoring gut barrier function. Anti-cytokine antibodies restore intestinal epithelial permeability by inhibiting epithelial cell apoptosis and maintaining expression and localisation of tight junction proteins. These therapeutic agents also reduce inflammatory responses by inhibiting bacterial translocation to lymph nodes, reducing leukocyte recruitment, as well as inhibiting colonic productions of proinflammatory cytokines. Majority of the anti-cytokine antibodies have beneficial effects on the gut barrier function; however, some of them such as anti-IL-17 antibody and IL-17 receptor antibody could cause exacerbation of colitis. TJ: tight junction; TNF: tumor necrosis factor; IL: interleukin; Ab: antibody; TGF*β*: transforming growth factor beta.

Antibodies blocking the activities of proinflammatory cytokines have been used in treating chronic inflammatory diseases and autoimmune disorders. Chronic inflammation and increased intestinal permeability are common clinical features observed in these diseases. In addition to the immunomodulatory effects, evidence reviewed in this paper suggests that anti-cytokine antibodies exhibit beneficial effects on the gut barrier function; the possible mechanisms that lead to such effects are summarised in Figure 1. In chronic inflammatory disorders treated with anti-cytokine antibodies, particularly IBD, these beneficial effects on the gut barrier function may contribute to the clinical improvements.

## Figures and Tables

**Table 1:** A list of clinically used anti-cytokine antibodies.

Therapeutic agent (trade name)	Cytokine target	Treated disease
Infliximab (Remicade)	TNF-*α*	Crohn's disease, ulcerative colitis, psoriatic arthritis, rheumatoid arthritis, plaque psoriasis, and ankylosing spondylitis
Adalimumab (Humira)		Crohn's disease, ulcerative colitis, psoriatic arthritis, rheumatoid arthritis, psoriasis, juvenile idiopathic arthritis, hidradenitis suppurativa, and uveitis
Certolizumab (Cimzia)		Crohn's disease, rheumatoid arthritis, psoriatic arthritis, ankylosing spondylitis, plaque psoriasis, and nonradiographic axial spondyloarthritis
Golimumab (Simponi)		Ulcerative colitis, rheumatoid arthritis, psoriatic arthritis, and ankylosing spondylitis
Canakinumab (Ilaris)	IL-1*β*	Cryopyrin-associated periodic syndromes, juvenile idiopathic arthritis, familial cold autoinflammatory syndrome, Muckle-Wells syndrome, and familial Mediterranean fever
Daclizumab (Zinbryta)	IL-2R	Multiple sclerosis
Dupilumab (Dupixent)	IL-4R	Atopic dermatitis and asthma
Mepolizumab (Nucala)	IL-5	Severe eosinophilic asthma and eosinophilic granulomatosis with polyangiitis
Reslizumab (Cinqair)		Severe eosinophilic asthma
Siltuximab (Sylvant)	IL-6	Castleman disease
Tocilizumab (Actemra)	IL-6R	Rheumatoid arthritis, juvenile idiopathic arthritis, giant cell arteritis, giant cell arteritis, and cytokine release syndrome
Sarilumab (Kevzara)		Rheumatoid arthritis
Guselkumab (Tremfya)	IL-23 p19 subunit	Plaque psoriasis
Tildrakizumab (Ilumya)		Plaque psoriasis
Ustekinumab (Stelara)	IL-12/IL-23 p40 subunit	Crohn's disease, psoriatic arthritis, and psoriasis
Ixekizumab (Taltz)	IL-17A	Plaque psoriasis and psoriatic arthritis
Secukinumab (Cosentyx)	IL-17A/F	Plaque psoriasis, ankylosing spondylitis, and psoriatic arthritis
Brodalumab (Siliq)	IL-17R	Plaque psoriasis

Only Food and Drug Administration approved therapeutic agents for clinical use are included. Data were obtained from URL: http://www.drugs.com (accessed on 08/04/2019).

**Table 2:** Increased intestinal permeability in chronic inflammatory and autoimmune diseases treated with anti-cytokine antibodies.

Diseases	Intestinal permeability (IP)	Permeability marker	Ref
IBD	Patients with CD and UC including those under remission had increased IP as compared with healthy controls.	Cellobiose/rhamnose; lactulose/rhamnose; cellobiose/mannitol; lactulose/mannitol; sucrose; sucralose; ^51^Cr-EDTA; 99mtc-diethylenetriaminopentaacetic acid; polyethylene glycol-400; iohexol	[110–127]
	Children with CD had increased IP as compared with healthy children.	Lactulose/mannitol	[128]
	Increased IP was detected prior to the onset of CD in an individual with familial risk.	^51^Cr-EDTA	[129]
	CD patients and their relatives had increased IP as compared with unrelated controls.	Polyethylene glycol-400 cellobiose/mannitol; lactulose/mannitol	[130] [131, 132]
	Higher IP was found in patients with CD and their spouses in comparison to controls.	Lactulose/mannitol	[133]

Psoriasis	Patients with psoriasis had increased IP as compared with healthy controls.	^51^Cr-EDTA	[134]

Rheumatoid arthritis	Patients with juvenile chronic arthritis displayed increased IP as compared with healthy controls.	Lactulose/mannitol	[135]
	A higher sucrose excretion but a normal lactulose/mannitol was found in patients with juvenile idiopathic arthritis as compared with controls.	Sucrose; lactulose/mannitol	[136]
	Patients with active rheumatoid arthritis had increased IP as compared with the control group.	Polyethylene glycol; ^51^Cr-EDTA	[137, 138]
	IP was found to be normal in untreated rheumatoid arthritis patients but abnormally increased in patients treated with nonsteroidal anti-inflammatory drugs.	^51^Cr-EDTA	[139]
	Patients with rheumatoid arthritis excreted less polyethylene glycol 400 and 1000 than healthy controls, whereas the excretion of polyethylene glycol 3000 was the same or greater than in healthy controls.	Polyethylene glycol 400, 1000, or 3000	[140]

Ankylosing spondylitis	Patients showed increased IP as compared to controls.	Polyethylene glycol; ^51^Cr-EDTA	[137, 141]
	IP was found to be higher in patients and their relatives as compared to controls.	^51^Cr-EDTA; lactulose; mannitol; sucrose	[142, 143]

IBD: inflammatory bowel disease; CD: Crohn's disease; UC: ulcerative colitis.

**Table 3:** Direct or indirect evidence of anti-cytokine antibodies acting on gut barrier function.

Anti-cytokine antibodies	Subjects	Effects of anti-cytokine antibodies	Ref
Ani-TNF Ab	SAMP1/YitFc mouse model with ileitis	(i) Downregulated epithelial apoptosis (ii) Decreased membrane bound Fas/CD95 expression (iii) Increased lamina propria mononuclear cell apoptosis	[28]
	Rat model of indomethacin-induced enterocolitis	(i) Prelesion administration of infliximab markedly reduced the intestinal permeability (ii) No obvious microscopic or macroscopic alterations	[29]
	Rat model of indomethacin-induced enterocolitis	(i) Alleviated small bowel inflammation partially due to improvement of increased intestinal permeability	[30]
	CD patients	(i) Increased intestinal permeability observed in patients before treatment has significantly reduced to levels within normal range	[25, 26, 144]
	Enteric biopsies of CD patients	(i) Reduced epithelial apoptosis (ii) Improved transepithelial electrical resistance	[27]

Anti-IL-6R Ab	SW480 cells	(i) Reversed the increased clonogenicity and invasiveness of SW480 cells induced by IL-6 (ii) Inhibited the release of invasion linked MMP-9 and MMP-2	[55]
	Mouse model of T cell transfer-induced colitis	(i) Reduced colitis score as comparing with the control group (ii) Reduced colonic mRNA levels of IFN-*γ*, TNF-*α*, and IL-1*β* (iii) Suppressed intercellular expressions of ICAM1 and VCAM1 in colonic vascular endothelial cells	[56]
	Mouse model of T cell transfer-induced colitis	(i) Reduced level of colitis (ii) Diminished leukocyte recruitment (iii) Increased T cell apoptosis	[57]
	CD patients	(i) An 80% therapeutic response was observed as compared with 30% response from the placebo group (ii) No obvious effect on mucosal healing	[54]

Anti-IL-6 Ab	Mouse model with ethanol- and burn-induced injury	(i) Reduced morphological changes in the ileum (ii) Decreased bacterial translocation to the mesenteric lymph node (iii) Reduced myosin light chain phosphorylation in intestinal epithelial cells (iv) Maintained ZO-1 and occludin localisation with actin	[58]
	Mouse sepsis model induced by caecal ligation and a puncture method	(i) Reduced serum productions of IL-6 and IL-10 (ii) Reduced colonic production of TNF-*α* (iii) Alleviated upregulation in mRNA expressions of E-cadherin and desmoglein-2	[59]
	Mouse model of DSS-induced colitis	(i) Reduced mucosal damages (ii) Decreased inflammatory infiltrates (iii) Improved intestinal permeability (iv) Suppressed expressions of claudin-2 and myosin light chain kinase	[71]

Anti-IL-12 Ab	IL-10-deficient mouse model of colitis	(i) Reduced colitis (ii) Reduced mesenteric lymph node and colonic CD4+ T cells (iii) Reduced IFN-*γ* producing T cells in the mesenteric lymph node	[77]
	Mouse model of TNBS-induced colitis	(i) Improved clinical and histological features of colitis (ii) Inability for lamina propria CD4+ T cells to secrete IFN-*γ* upon *in vitro* stimulation	[78]
	MRL/MpJ-lpr^fas^ mice and SJL/J mice with TNBS colitis	(i) Anti-IL-12 antibody induced T cell apoptosis through Fas pathway	[79]
	CD patients	(i) Decreased productions of IL-12, IFN-*γ*, and TNF-*α* in colonic lamina propria mononuclear cells (ii) No significant difference in the remission rate was observed between the treatment group and the placebo group	[80]

Anti-IL-23 Ab	Mouse model of TNBS-induced colitis	(i) Reduced colitis (ii) Restored downregulation of claudin-8 in colonic mucosa	[82]
	CD patients	(i) Higher remission rate as compared with the placebo group (ii) Reduced levels of biomarkers C-reactive protein and faecal calprotectin	[88]
	Enteric biopsies of CD patients	(i) Decreased colonic expression of genes associated with the IL-23/IL-17 axis such as IL-23, IL-26, and IL-17A	[89]

Anti-p40-Ab	CD patients	(i) Induced clinical response	[90, 91]

Anti-IL-17 Ab	Mouse model of DSS-induced colitis	Anti-IL-17 Ab (i) Increased mucosal mRNA levels of TNF-*α*, IFN-*γ*, IL-6, RANTES, and IP-10	[103]
	Mouse model of DSS-induced colitis	Pretreatment with anti-IL-17F Ab (i) Suppressed inflammation induced caecal edema and colonic shortening	[145]
	Multidrug resistance mouse model of colitis	Inhibition with anti-IL-17A or anti-IL-17RA Ab (i) Promoted colitis exacerbation (ii) Severe weakening of the intestinal epithelial barrier (iii) Enhanced colonic inflammation	[104]
	Mouse model of T cell transfer-induced colitis	Coinhibition with anti-IL-17A and IL-17F Ab (i) Reduced intestinal inflammation	[105]
	CD patients	Inhibition with anti-IL-17A Ab (i) Adverse events (ii) Elevated inflammatory markers including C-reactive protein and faecal calprotectin	[107]

Ab: antibody; CD: Crohn's disease; IL: interleukin; MMP: matrix metalloproteinase; VCAM: vascular cell adhesion molecule; ICAM: intercellular adhesion molecule; IFN: interferon; IP-10: IFN-*γ*-induced protein-10; TNF: tumor necrosis factor; ZO: zonula occluden; STAT: signal transducer and activator of transcription; HLA: human leukocyte antigen.

## Abbreviations

CD: Crohn's disease
DSS: Dextran sulfate sodium
HLA-DR: Human leukocyte antigen-DR
IBD: Inflammatory bowel disease
ICAM: Intercellular adhesion molecule
IL: Interleukin
IP-10: IFN-*γ*-induced protein-10
KC: Keratinocyte chemoattractant
MCP: Monocyte chemoattractant protein
MLCK: Myosin light chain kinase
MMP: Matrix metalloproteinase
NF-*κ*B: Nuclear factor kappa B
STAT: Signal transducer and activator of transcription
TGF*β*: Transforming growth factor beta
TJ: Tight junction
TNBS: 2,4,6-Trinitrobenzenesulfonic acid
TNF: Tumor necrosis factor
UC: Ulcerative colitis
VCAM: Vascular cell adhesion molecule
ZO: Zonula occluden.
